# Progranulin deficiency in the brain: the interplay between neuronal and non-neuronal cells

**DOI:** 10.1186/s40035-025-00475-8

**Published:** 2025-04-16

**Authors:** Katarzyna Gaweda-Walerych, Vanessa Aragona, Simona Lodato, Emilia J. Sitek, Ewa Narożańska, Emanuele Buratti

**Affiliations:** 1 Department of Neurogenetics and Functional Genomics, Mossakowski Medical Research Institute, Polish Academy of Sciences, 02-106 Warsaw, Poland; 2 Department of Biomedical Sciences, Humanitas University, Via Levi Montalicini 4, Pieve Emanuele, 20072 Milan, Italy; 3 Neurodevelopment Biology Lab, IRCCS Humanitas Research Hospital, via Manzoni, 56, Rozzano, 20089 Milan, Italy; 4 Division of Neurological and Psychiatric Nursing, Laboratory of Clinical Neuropsychology, Neurolinguistics, and Neuropsychotherapy, Faculty of Health Sciences, Medical University of Gdansk, 80-210 Gdansk, Poland; 5 Neurology Department, St. Adalbert Hospital, Copernicus PL, 80-462 Gdansk, Poland; 6 Molecular Pathology Group, International Centre for Genetic Engineering and Biotechnology (ICGEB), AREA Science Park, 34149 Trieste, Italy

**Keywords:** Progranulin deficiency, TAR-DNA binding protein 43, Frontotemporal dementia, Primary progressive aphasia, Astrocytes, Microglia, Oligodendrocytes, Neurovasculature, Intercellular communication, Lipid dyshomeostasis

## Abstract

Heterozygous mutations in *GRN* gene lead to insufficient levels of the progranulin (PGRN) protein, resulting in frontotemporal dementia (FTD) with TAR DNA-binding protein 43 (TDP-43) inclusions, classified pathologically as frontotemporal lobar degeneration (FTLD-TDP). Homozygous *GRN* mutations are exceedingly rare and cause neuronal ceroid lipofuscinosis 11, a lysosomal storage disease with onset in young adulthood, or an FTD syndrome with late-onset manifestations. In this review, we highlight the broad spectrum of clinical phenotypes associated with PGRN deficiency, including primary progressive aphasia and behavioral variant of frontotemporal dementia. We explore these phenotypes alongside relevant rodent and in vitro human models, ranging from the induced pluripotent stem cell-derived neural progenitors, neurons, microglia, and astrocytes to genetically engineered heterotypic organoids containing both neurons and astrocytes. We summarize advantages and limitations of these models in recapitulating the main FTLD-*GRN* hallmarks, highlighting the role of non-cell-autonomous mechanisms in the formation of TDP-43 pathology, neuroinflammation, and neurodegeneration. Data obtained from patients’ brain tissues and biofluids, in parallel with single-cell transcriptomics, demonstrate the complexity of interactions among the highly heterogeneous cellular clusters present in the brain, including neurons, astrocytes, microglia, oligodendroglia, endothelial cells, and pericytes. Emerging evidence has revealed that PGRN deficiency is associated with cell cluster-specific, often conserved, genetic and molecular phenotypes in the central nervous system. In this review, we focus on how these distinct cellular populations and their dysfunctional crosstalk contribute to neurodegeneration and neuroinflammation in FTD-*GRN*. Specifically, we characterize the phenotypes of lipid droplet-accumulating microglia and alterations of myelin lipid content resulting from lysosomal dysfunction caused by PGRN deficiency. Additionally, we consider how the deregulation of glia-neuron communication affects the exchange of organelles such as mitochondria, and the removal of excess toxic products such as protein aggregates, in PGRN-related neurodegeneration.

**Supplementary Information:**

The online version contains supplementary material available at 10.1186/s40035-025-00475-8.

## Background

Progranulin (PGRN) is a multifunctional glycoprotein expressed throughout the body. PGRN deficiency is particularly harmful to the central nervous system (CNS) [1, 2]. *GRN* mutations were first identified in 2006 as a cause of frontotemporal dementia (FTD) with TAR DNA-binding protein 43 (TDP-43) inclusions [3–5]. Six years later, they were identified to be also linked to neuronal ceroid lipofuscinosis 11 (CLN11), a rare lysosomal storage disorder [6], expanding the spectrum of *GRN*-related neurodegenerative disorders.

Mouse models of Pgrn deficiency and single-cell models derived from iPSCs of FTD patients have provided valuable insights, but they do not fully recapitulate phenotypes or disease hallmarks (such as TDP-43 pathology) observed in patients [7–10]. Recently developed engineered human brain organoids, composed of PGRN-deficient neurons or astrocytes, have shown improved ability to model TDP-43 pathology, highlighting the importance of non-cell-autonomous mechanisms [11].

Recent studies using single-cell transcriptomics have documented that all brain cell types, including neurons, astrocytes, microglia, oligodendroglia, endothelial cells, and pericytes, are impacted by PGRN deficiency. This deficiency contributes to lysosomal deregulation, protein and lipid dyshomeostasis, synaptic dysfunction, neuroinflammation, and demyelination in a cell type-dependent manner [7, 11–18].

Various PGRN-deficient glial cell populations show specific transcriptomic and metabolic/inflammatory signatures, morphological alterations, and impaired phagocytic capabilities [7, 12–15].

Neuroinflammation, characterized by glial activation and release of inflammatory mediators, is a common feature of virtually all neurodegenerative diseases [19–21]. Specific neuroinflammatory markers or their combinations have been reported in FTD-*GRN* patients and PGRN deficiency models [12, 22–24]. Pathways involved in the protective and detrimental effects of neuroinflammation in PGRN deficiency are also being revealed [13, 15, 16].

In addition, emerging studies modeling the bidirectional exchange of organelles and metabolism products (such as TDP-43 aggregates and lipids) in cocultures of neurons and different types of glia [25–27] can delineate important yet lesser-known aspects of the interplay between CNS cellular clusters in the context of PGRN deficiency. However, the effect of PGRN deficiency on the integrity of brain barriers, such as the blood–brain barrier (BBB) [28, 29] or the blood-cerebrospinal fluid barrier (BCSFB), has only begun to be addressed.

For this review, we searched PubMed and Medline databases using search term “progranulin (PGRN or GRN)”, combined with “TAR-DNA binding protein 43 (TDP-43)”, “protein aggregate”, “frontotemporal (lobar) degeneration (FTLD, FTD)”, “neuronal ceroid lipofuscinosis-11 (CLN11)”, “primary progressive aphasia (PPA)”, “behavioral variant frontotemporal dementia (bvFTD)”, “neuroinflammation”, “neuron”, “synapse pruning”, “astrocyte”, “microglia”, “oligodendrocyte”, “vasculature”, “complement”, “wingless-type mmtv integration site family (WNT)”, “lipid”, “transcriptomics”, “lipidomics”, “proteomics”, “induced pluripotent stem cells (iPSCs)”, “i-neurons”, “i-astrocytes”, “i-microglia”, “organoid”, “mitochondria”, “transmitophagy”, “lysosome”, “autophagy”, “mitophagy”, “exosome”, “cerebrospinal fluid (CSF)”, “BBB”, and “choroid plexus (ChP)”. Most of the papers reviewed were published within the recent 10 years.

## Structure and function of PGRN and the molecular mechanisms underlying its deficiency

PGRN is a ubiquitously expressed intracellular and secreted glycoprotein that acts as a multifunctional growth factor with neurotrophic properties, participating in cell growth/survival, embryogenesis, wound repair, endolysosomal homeostasis, autophagy, and inflammation [2, 30–32]. *GRN* mutations are linked to neurodegeneration through lysosome malfunctioning, neuroinflammation, synaptic dysfunction, and TDP-43 pathology [1, 30, 33–35].

One of the indicators of PGRN involvement in endolysosomal homeostasis is the detection of CLN11 hallmarks (i.e., accumulation of lipofuscin, saposin D, cathepsin D, lysosomal transmembrane protein 106B [TMEM106B], and LAMP1/2) in the brains, skin, and retinas of FTD-*GRN* patients [36, 37]. Since then, lysosomal dysfunction due to PGRN deficiency has been increasingly studied [14, 15, 35, 38–45] and reviewed extensively [30, 33, 35, 46].

PGRN can enter lysosomes via sortilin-mediated endocytosis or be targeted into lysosomes by prosaposin (PSAP), via mannose 6-phosphate receptor or low-density lipoprotein receptor-related protein 1 [34, 47–49]. In the lysosomal compartment, PGRN (comprising 7.5 granulin repeats) is proteolytically cleaved into individual granulins: P, G, F, B, A, C, D, and E [50], by various cathepsins (L, B, E, G, K, S, V) and asparagine endopeptidase (AEP) in a pH-dependent manner [39, 51–53]. Multiple granulin fragments are also generated [53]. The levels of individual granulin peptides are differentially regulated and affected by PSAP and various cathepsins [54].

Like PGRN, multiple granulins are also haploinsufficient in primary fibroblasts and cortical brain tissues from FTD-*GRN* patients [39], accompanied by increased PGRN processing to granulin F and elevated AEP activity in degenerating regions [53]. Recent findings have highlighted the therapeutic potential of human granulin peptides, as their expression can ameliorate lysosome dysfunction, lipid dysregulation, microgliosis, and lipofuscinosis in *Grn*^−/−^ mice as efficiently as full-length PGRN [44].

Multiple studies have demonstrated that PGRN deficiency leads to the upregulation of lysosomal gene expression and protein level, impaired clearance of autophagosomes, and TDP-43 aggregation in the brains of FTD-*GRN* patients and in mouse and cellular models [14, 16–18, 36, 38, 40, 44, 55–57]. PGRN regulates lysosomal acidification [40, 57], which is essential for the optimal activity of luminal enzymes (usually between 4.5 and 5.0). Indeed, in iPSC-derived glutamatergic neurons, PGRN loss leads to increased lysosomal pH which reduces the degradative capacity of the lysosomes, and this may be compensated for by increases in hydrolases and vacuolar-type ATPase subunits responsible for lysosomal acidification [56–58]. In addition, PGRN and granulins regulate activities of enzymes such as cathepsin D and glucocerebrosidase (GCase) in the lysosome [15, 44, 45, 59–62]. FTD-*GRN* brains and iPSC-derived neurons with *GRN* mutations demonstrate impaired processing of PSAP to saposin C (a critical activator of GCase), resulting in reduced GCase activity, providing insight into the link with lysosomal storage disorders [63]. Indeed, the mutual interactions between PSAP and PGRN can impact their lysosomal trafficking [64–66].

Another important partner/modifier of PGRN function is the TMEM106B, revealed by genetic association studies on FTD-*GRN* patients [67–70]. Similar to PGRN, TMEM106B regulates lysosomal pH and function of lysosomal proteins [69, 71]. TMEM106B accumulates in FTD-*GRN* brains and *Grn*^*−/−*^ mice [72], as reviewed in [35], and its increased expression confers increased disease risk [73, 74], suggesting that lowering TMEM106B might be a therapeutic strategy. *Tmem106b*^*−/−*^ mice display a mild motor phenotype with subtle astrocyte activation [75]. However, further research consistently showed that *Grn*/*Tmem106b* double knockouts (DKO) develop severe phenotypes, characterized by motor deficits, premature death, neurodegeneration, glial activation, lysosomal abnormalities, and phospho-Tdp-43 pathology, with a much earlier onset than *Grn*^−/−^ mice [35, 71, 76–79]. Accordingly, TMEM106B deletion in PGRN-deficient iPSC-derived human microglia did not normalize transcriptomic or proteomic profiles [79]. The initially observed phenotype amelioration in *Grn/Tmem106b* DKOs [80] is attributed to residual Tmem106b levels in this model [71]. Altogether, these results indicate that lowering TMEM106B level is not a viable therapeutic strategy for treating FTD-*GRN.*

## PGRN deficiency spectrum leading to diverse clinical entities

**Fig. 1:**
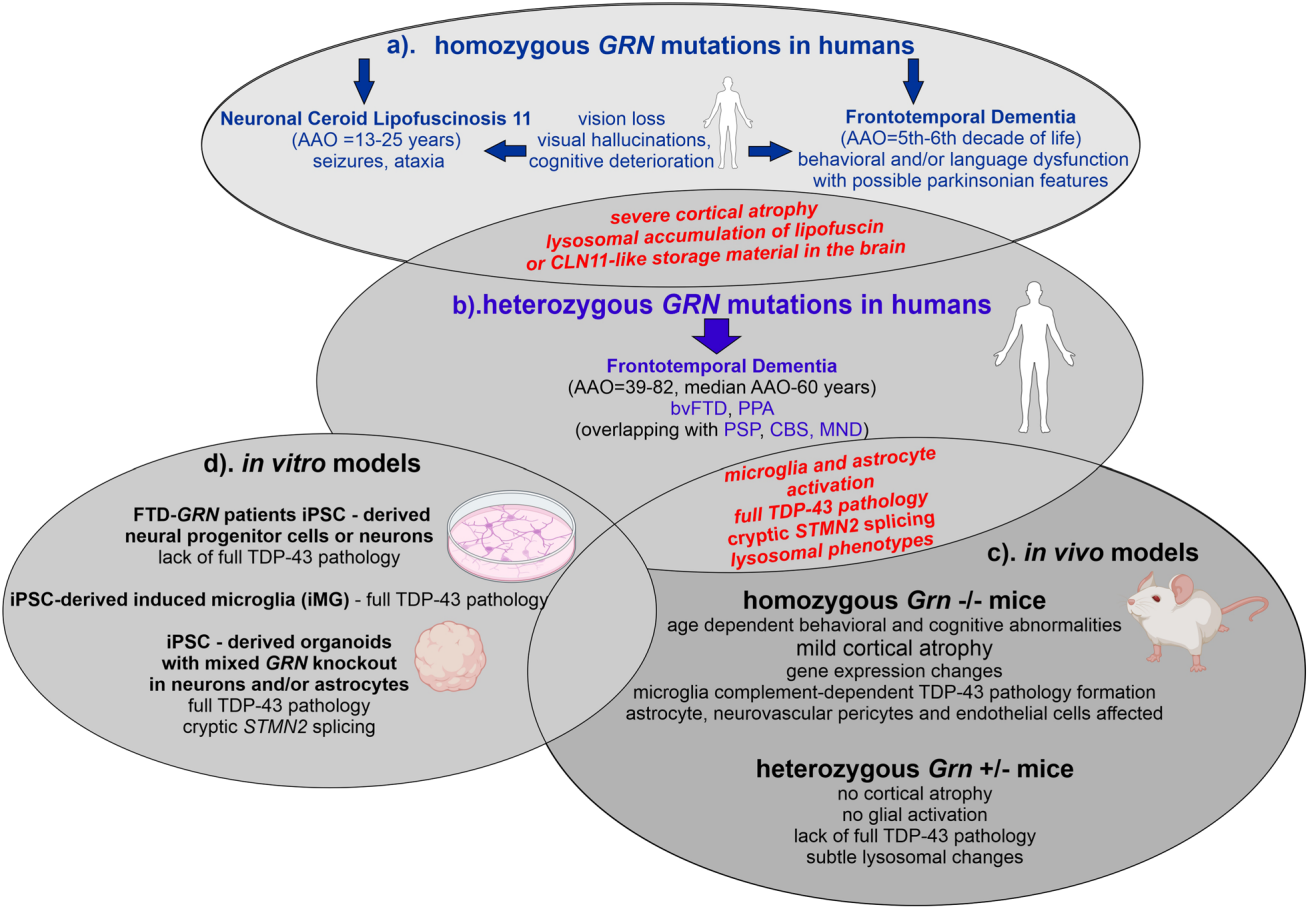
Comparison of patients carrying *GRN* mutations with rodent- and human-derived models of PGRN deficiency. **a** In humans, homozygous *GRN* mutations are extremely rare and cause CLN11 or FTD syndromes [6, 37, 88, 118, 119]. **b** FTD caused by heterozygous *GRN* mutations (FTD-*GRN*) is characterized by severe cortical atrophy [120], full TDP-43 pathology [4, 121–124]; microgliosis [14, 125, 126], astrogliosis [7, 12, 91, 115, 127], and lysosomal phenotypes [128, 129]. The full TDP-43 pathology includes nuclear depletion, cytoplasmic hyperphosphorylated and ubiquitinated inclusions, and downstream loss-of-function phenotypes, such as cryptic splicing of *STMN2* and *UNC13a*. **c** In vivo models of Pgrn deficiency. *Grn*^−/−^ mice reproduce key clinical and neuropathological features characteristic of FTD-*GRN* patients, i.e., age-dependent behavioral/cognitive abnormalities, microglia and astrocyte activation, and TDP-43 pathology [16, 36, 130–132]. Cortical atrophy and TDP-43 proteinopathy are more accentuated in patients than in animal models [7]. Hemizygous mice (*Grn*^+/−^) show milder phenotypes than FTD-*GRN* patients carrying heterozygous *GRN* mutations, with no TDP-43 pathology or significant glial activation [36, 133–135]. **d** In vitro human-derived cellular models of PGRN deficiency. In human iPSC-derived neurons or neuronal progenitor cells, the presence of full TDP-43 pathology is debated [9, 56, 136]. In contrast, induced microglia (iMG) with *GRN* knockout display cytoplasmic TDP‑43 aggregation, a severe neuroinflammation phenotype, impaired phagocytosis, and a disease-associated signature [14, 15]. Human iPSC-derived organoid-like cocultures with *GRN* knockout in neurons or astrocytes recapitulate full TDP-43 pathology [7, 11]. AAO, age at onset; bvFTD, behavioral variant of frontotemporal dementia; CBS, corticobasal syndrome; CLN11, neuronal ceroid lipofuscinosis 11; FTD-*GRN,* frontotemporal dementia caused by *GRN* mutations; PGRN, progranulin; iMG, induced microglia; iPSC, induced pluripotent stem cells; MND, motor neuron disease; PPA, primary progressive aphasia; PSP, progressive supranuclear palsy; *STMN2,* stathmin-2; TDP-43, TAR-DNA binding protein 43; *UNC13a,* unc −13 homolog A

FTD is a clinical syndrome characterized by progressive changes in behavior, personality, language, and motor skills due to the degeneration of the frontal and temporal lobes of the brain [81]. FTD is the second most common cause of dementia in populations under 65 years of age, with prevalence between 0.01 and 4.6 per 1000 persons [82, 83]. Approximately 30%–40% of FTD patients have a family history, most commonly caused by pathogenic variants in *MAPT, C9orf72*, and *GRN* [81]. Heterozygous *GRN* mutations account for approximately 13.9% of FTD patients [84]. Rare homozygous loss-of-function mutations of *GRN* cause CLN11 with early adulthood onset [6], but some also lead to a unique FTD phenotype [37] (Fig. 1).

Over 130 pathogenic *GRN* variants have been identified, predominantly leading to premature termination of PGRN protein synthesis through nonsense-mediated mRNA decay [85, 86]. This results in PGRN haploinsufficiency in FTD patients [8, 87] or complete absence in CLN11 patients [6, 37, 88]. PGRN levels are typically decreased by 50%–70% in various body fluids or cells in FTD-*GRN* patients [8, 87, 89–93]. In CLN11 patients, PGRN is usually undetectable, but with rare splicing mutations, some functional PGRN is still produced [6, 37, 88]. Hence, *GRN* premature termination variants are highly pathogenic while rare splicing and missense mutations have variable effects on protein function [94]. PGRN is involved in various neurodegenerative diseases, such as Alzheimer’s disease (AD), rare amyotrophic lateral sclerosis–frontotemporal dementia spectrum disorder (ALS-FTSD) phenotypes [95], and certain cancers, where increased PGRN expression promotes disease progression [30, 96, 97]. Therefore, tight regulation of PGRN levels is crucial. Genome-wide association studies have linked *GRN* variants to AD, limbic-predominant age-related TDP-43 encephalopathy, and ALS-FTSD caused by hexanucleotide repeat expansion in the *C9orf72* gene [98–101].

### PGRN expression in neuronal and non-neuronal cells in the CNS

At the molecular level, *GRN* gene expression is regulated by TFEB (transcription factor EB), the master transcription factor of lysosomal biogenesis, autophagy, and lipid catabolism [102]. PGRN is widely expressed in the human body. Initial research focused on its role in angiogenesis, vascular inflammation, and endothelial cell function [103–106]. The adult mouse brain shows robust Pgrn expression in the neocortex and hippocampus; moderate expression in the thalamus, hypothalamus, amygdala, and midbrain; and low expression in the striatum/brainstem [107]. Regarding CNS cell types, initial data revealed robust Pgrn expression in microglia [107]. Subsequent research in mice and humans revealed significant astrocytic expression and secretion of PGRN [7, 41, 108, 109]. Single-nucleus RNA sequencing (snRNAseq) studies have mapped *GRN* expression in different cell clusters of the mouse and human brains [7, 13], showing the most abundant expression in microglia. Other cell clusters, such as neurons, astrocytes, oligodendroglia, or oligodendroglial precursors, present relatively low *GRN* mRNA expression [7]. Interestingly, wild-type hiPSC-derived astrocytes display significantly greater expression than neurons [108]. In addition, granulins exhibit neuronal and microglial localization (specifically granulin A in the lysosomal compartment), while the highest ratio between granulins and PGRN is observed in the cortical region of the mouse brain [54].

Both peripheral [104, 110] and CNS glial expression of PGRN and granulins is strongly stimulated during inflammation [54], modulated by secretory leukocyte protease inhibitor (SLPI) [109, 111]. Interestingly, high SLPI levels affect the penetrance of FTD-*GRN* by delaying disease onset [112]. Viral infections also upregulate neuronal PGRN expression [113], indicating another possible connection between TDP-43 and viral infections, as recently reviewed [114]. Interestingly, PGRN expression does not directly correlate with the gray matter atrophy patterns in symptomatic FTD patients' brains [115], suggesting complex regulatory mechanisms beyond PGRN levels alone.

Previous research demonstrated that reduction of microglial Pgrn does not exacerbate behavioral phenotypes or pathology in neuronal Pgrn-deficient mice [116]. In line with these findings, selective neuronal expression of Pgrn is sufficient to rescue the structural change and inflammation after traumatic brain injury (TBI) in full *Grn*-knockout mice [117]. Notably, infiltration of CD68^+^ microglia was reduced. These findings highlight the importance of PGRN expression and function in neurons and raise the possibility of cross-correction between CNS cell types with important implications for therapeutic approaches.

### Brain regions affected in FTD-*GRN* patients

In contrast to the wide range of clinical manifestations accompanying *GRN* mutations, such as changes in behavior, executive functions, and/or language [137], the associated pathology, termed frontotemporal lobar degeneration (FTLD), is relatively homogeneous, with cortical atrophy tending to be most severe in the frontal lobes [120, 138]. Additionally, atrophy of the parietal lobe and neuronal loss in the substantia nigra occur more frequently in FTD-*GRN* patients than in non-*GRN* FTD patients [95]. Initial symmetrical atrophy becomes asymmetrical later in the disease course. Presymptomatic *GRN* mutation carriers exhibit isolated gray matter density loss in the orbitofrontal and occipital cortex [139], whereas in symptomatic patients, atrophy is more widespread. Cerebral hypometabolism appears 7–25 years before the onset of clinical symptoms [140]. Similar to symptomatic individuals, presymptomatic *GRN* carriers show discrete regions of hypometabolism in the right anterior cingulate, insula and orbitofrontal cortex [141]; left lateral temporal lobe [142]; and frontal, parietal, and hippocampal regions [143]. However, regions of hypometabolism cannot be correlated with TDP-43 pathology in vivo, as there is no dedicated positron emission tomography (PET) tracer [144]. The pattern of progression indicates early anterior changes, which later generalize to posterior regions and are not always asymmetric [145]. In some cases, FTD-*GRN* pathology also affects regions of the limbic system, such as the hippocampus and amygdala, contributing to the emotional and memory-related disturbances [146]. In some cases, cerebellar regions and deep subcortical structures may also be affected [147–149].

### Main symptoms, characteristics and genetic modifiers of FTD-***GRN*** patients

The spectrum of clinical presentations associated with mutations in *GRN* is highly heterogeneous, even among family members carrying the same mutation, making genotype‒phenotype correlations difficult [150]. The age of onset may also vary considerably within families with *GRN* mutations, ranging from 39 to 89 years, with a median age of onset of 60 years [68, 151, 152], and disease duration ranges from 3 to 22 years [147]. *GRN* mutations are not fully penetrant. In some cases, carriers may develop clinical symptoms even in their 90 s [153]. In addition to the Mendelian genetic factors in FTD, additional genetic, epigenetic, and environmental factors might modify the phenotypic presentation of the disease. Among the most extensively studied genetic modifiers to date, *TMEM106B* stands out as the strongest genetic modifier [67–70]. In the largest study on genetic modifiers in FTD-*GRN*, carriers of the *TMEM106B* protective haplotype (‘G’ allele of rs3173615) had 50% lower odds of developing disease symptoms compared to non-protective haplotype carriers [68]. Another study reported another protective minor allele of *TMEM106B,* rs1990622, in association with greater gray matter volume in FTD-*GRN* brains, especially in the left thalamus [154]. Thus, the authors recommended routine *TMEM106B* genotyping alongside *GRN* genetic testing. Other genetic modifiers include *PSAP* [66], *GFRA2* [68, 155] and *FAM171A2* [156]. A meta-analysis examining sex differences revealed a 33% greater prevalence of FTD-*GRN* in females, suggesting that sex-related risk factors may moderate the expression of the disease phenotype [157]. Further research confirmed that *GRN* mutations are more prevalent in women (58.4%) than in men (41.6%) [85].

**Fig. 2:**
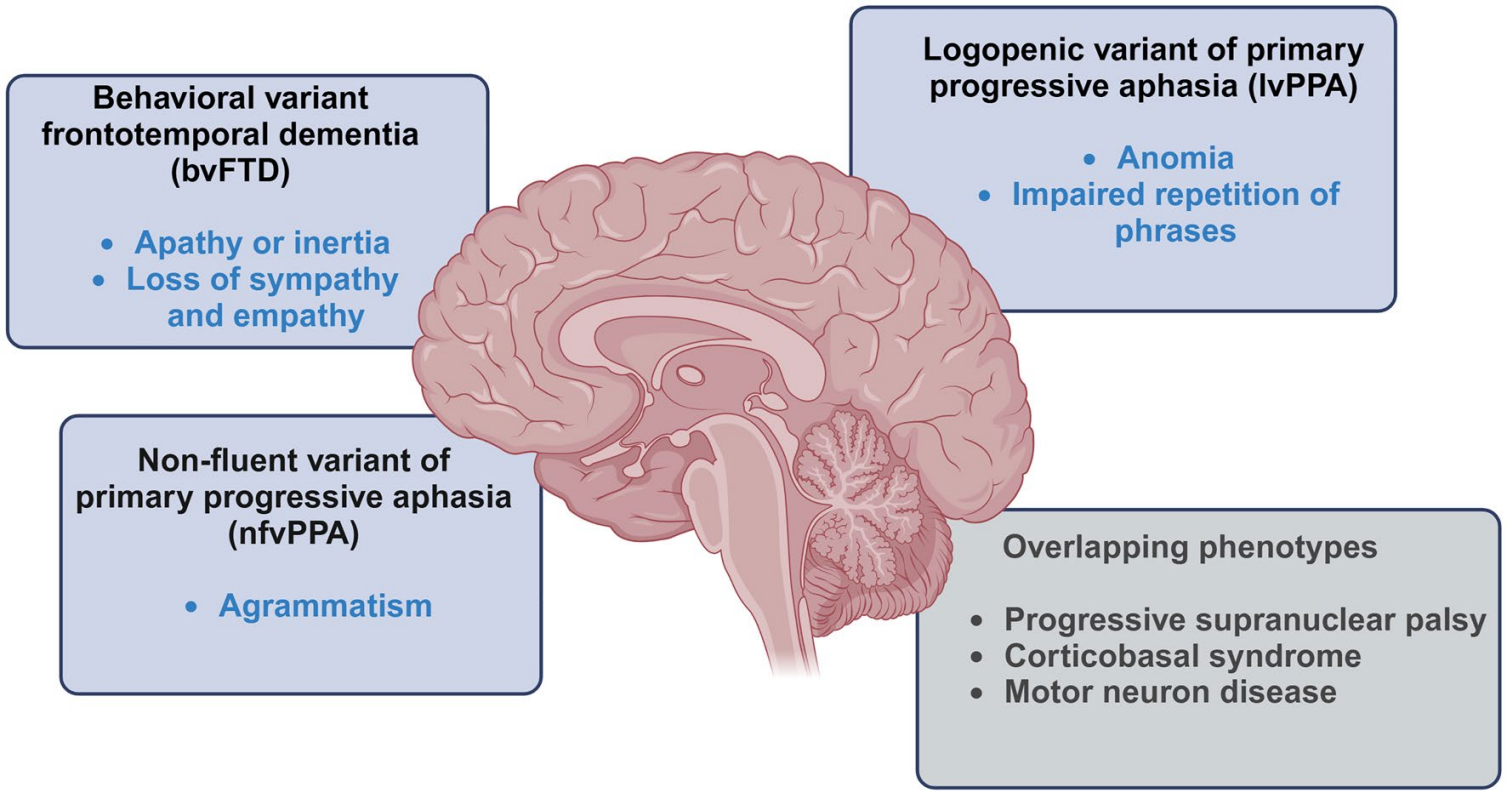
The spectrum of clinical presentations of FTD-*GRN*. Predominant clinical phenotypes of FTD-*GRN* include non-fluent (nfvPPA) and logopenic variants of primary progressive aphasia (lvPPA) and, less frequently, behavioral-variant frontotemporal dementia (bvFTD) [81]. Other phenotypes observed in FTD-*GRN* patients include progressive supranuclear palsy, corticobasal syndrome, and motor neuron disease [96, 161]

The spectrum of clinical presentations of FTD-*GRN* includes abnormalities in behavior and personality, language deficits, limb apraxia (the loss of ability to carry out learned purposeful movements, independent of sensory‒motor impairments and other cognitive deficits) [158], and parkinsonism [37]. Generally, FTD-*GRN* presents with two main clinical phenotypes: PPA and bvFTD [81] (Fig. 2). Interestingly, while bvFTD is the most common subtype of FTD, the most prevalent manifestation of FTD-*GRN* is PPA, especially progressive non–fluent variant of PPA (nfvPPA), also known as progressive non–fluent aphasia [81]. Parkinsonism is observed in approximately 40% of cases [143, 159], whereas episodic memory impairment, suggestive of AD-like phenotype, occurs in 10%–30% of cases [93, 160].

Overall, nfvPPA is characterized by poor expressive language, including hesitant and effortful speech with phonemic errors, grammatical impairment, and word-finding difficulties (concerning mainly, but not exclusively, verbs). The apraxia of speech may be accompanied by unclear speech (dysarthria), which may progress to anarthria (the inability to produce clear, articulate speech). Sentence comprehension is impaired, while word comprehension typically remains intact [162]. Patients with the nfvPPA phenotype due to *GRN* mutations exhibit more prominent anomia (word-finding problems) (Fig. 2 and Table S1) than other nfvPPA patients do, resembling the language difficulties observed in the logopenic variant of PPA, which is typically associated with AD [158, 163]. Speech apraxia (articulatory problems) is uncommon in nfvPPA cases caused by *GRN* mutations [87] and TDP-43 pathology, as it is usually associated with tau pathology [81]. Agrammatic speech without speech apraxia is predictive of *GRN* mutation [164] (Fig. 2 and Table S1). However, limb apraxia, the inability to perform skilled or learned limb actions on request or imitation, is common among carriers of *GRN* mutations [165]. Dyscalculia has also been described [166].

In bvFTD, core behavioral features include social disinhibition resulting from reduced awareness of social conventions and normative behaviors; apathy; repetitive, obsessive, and stereotyped behaviors; and dietary changes, such as altered preferences for sweet foods. Additionally, hyper- or hyposensitivity to pain has also been reported [167]. In bvFTD patients with *GRN* mutations, apathy predominates (Fig. 2 and Table S1), in contrast with the prominent disinhibition and ritualistic behaviors associated with *MAPT* mutations [159]. Patients with *GRN* mutations tend to be withdrawn and are unlikely to spontaneously initiate activity. Emotional blunting (the inability to fully experience both positive and negative emotions) may be pronounced (Table S1). Some FTD-*GRN* patients may experience psychotic symptoms such as delusions and hallucinations [168]. Among heterozygous *GRN* mutation carriers, complex visual hallucinations (people and animals) are quite frequent (25%) [143]. Executive dysfunction disrupts the ability of FTD patients to plan and engage in purposeful activities [169, 170]. Furthermore, *GRN* mutation carriers may exhibit a phenotype resembling AD, not only in terms of language presentation at the clinical stage but also in marked episodic memory impairment as well as early visuospatial and working memory deficits [143, 171].

The earliest neuropsychological manifestations in *GRN* mutation carriers include impaired facial emotion recognition [172, 173] as well as attention and executive deficits [174]. In a recent study carried out by the Genetic Frontotemporal Dementia Initiative (GENFI, http://www.genfi.org), the neuropsychological characteristics of FTD-*GRN* patients are correlated with neurotransmitter deregulation [175]. Specifically, magnetic resonance imaging (MRI) voxel-based brain changes reflecting atrophy patterns correlate with the spatial distribution of receptors for dopamine, serotonin, glutamate and acetylcholine. Importantly, these neurotransmitter alterations are observed in symptomatic but not in asymptomatic *GRN* mutation carriers. Loss of empathy and poor response to emotional cues are negatively associated with dopamine receptors (D1 receptors), and D1 receptors and dopamine transporters, respectively [175]. Intriguingly, long-term potentiation-like plasticity defects are evident even > 15 years before expected symptom onset in presymptomatic carriers of *GRN* mutations compared with neurologically healthy controls [175].

Motor neuron disease is rare in FTD-*GRN* mutation carriers [161, 176]. The most common motor symptom in carriers of *GRN* pathogenic variants is parkinsonism, which is characterized by rest and postural tremors and rigidity [161]. Parkinsonian features, such as slowness of movement, muscle rigidity, and postural instability, are more likely to occur in FTD-*GRN* than in FTD-TDP without *GRN* mutations [152, 161, 177]. We have previously discussed the co-occurrence of parkinsonism in FTD-TDP patients, including FTD-*GRN* patients [178]. Signs of corticobasal syndrome (CBS), including cortical sensory loss, limb apraxia, and alien limb phenomenon, are slightly more common in carriers of *GRN* pathogenic variants than are signs of progressive supranuclear palsy (PSP), such as neck rigidity, impaired eyelid function, supranuclear gaze palsy, dystonia, pseudobulbar palsy, and ataxia [161]. Clinical symptoms may sometimes gradually progress from typical clinical features of bvFTD to PSP-like symptoms, including dysarthria, dysphagia, and vertical supranuclear gaze palsy [96].

All the symptoms described above pertain to FTD cases caused by heterozygous mutations in *GRN*. Homozygous *GRN* mutations are exceedingly rare and, as mentioned above, result in lysosomal storage disease known as CLN11, which has been identified in only a few patients [6, 88]. This rare disorder is clinically characterized by a different set of symptoms than FTD-*GRN*, namely, cerebellar ataxia, seizures, vision loss (retinitis pigmentosa), and cognitive dysfunction, with onset typically occurring between 13 and 25 years of age (Fig. 1). However, in 2020, a study reported six patients carrying homozygous *GRN* mutations with later onset. These patients presented a less severe neurological phenotype consistent with bvFTD ± parkinsonism occurring after the age of 50 [37]. None of them had developed other cardinal features of CLN11, such as cerebellar ataxia, cerebellar atrophy, or epilepsy. Visual hallucinations were present in CLN11 and FTD-*GRN*, illustrating a clinical continuum between these entities [37]. This highlights the importance of *GRN* dosage, which depends on the type of *GRN* mutation and modifying factors (Fig. 1).

### TDP-43 pathology in FTD-*GRN* vs CLN11 patients

The neuropathology in FTD-*GRN* is characterized by frontotemporal lobar degeneration with TDP-43 inclusions (FTLD-TDP) [1]. Different TDP-43 inclusions are defined based on their shape, distribution, and cellular localization, and only recently has their structure been revealed via cryo-electron microscopy [179]. Among the distinct subtypes (A-E) of FTLD-TDP pathology, FTD-*GRN* patients exhibit exclusively the type A pathology, characterized by neuronal cytoplasmic inclusions and short dystrophic neurites [153, 180–183]. At present, it is still not clear why *GRN* mutations always induce type A pathology. The mechanism may be, at least partially, attributed to the presence of specific posttranslational modifications of TDP-43, such as phosphorylation, N-terminal acetylation, and deamidation, as recently identified via mass spectrometry analysis [184, 185]. Intriguingly, the formation of pathological TDP-43 C-terminal fragments in PGRN deficiency is specifically linked to the increased activity of lysosomal protease legumain [186]. The typical TDP-43 pathology observed in affected brain regions of FTD-*GRN* consists of TDP-43 nuclear depletion and hyperphosphorylated, polyubiquitinated inclusions in the cytoplasm [4, 5, 187–189]. In normal healthy cells, TDP-43 is a major regulator of all aspects of the RNA life cycle, particularly pre-mRNA splicing. As a consequence, its nuclear depletion leads to a multitude of splicing defects, such as inclusions of cryptic exons [122, 123]. Accordingly, mis-splicing of *STMN2* transcripts has recently become an established marker of TDP-43 pathology and is correlated with the level of phospho-TDP-43 pathology in the brains of patients [122, 190, 191].

Apart from TDP-43 pathology, there is also an increased aggregation of the TMEM106B C-terminal fragment [192], which forms amyloid fibrils in the brains of patients with FTLD-TDP and other neurodegenerative conditions and in normal aging brains [192–194].

The lack of typical TDP-43 pathology (absence of phosphorylated TDP-43 aggregates) in the brains of CLN11 patients, who carry homozygous *GRN* mutations, suggests (a) a dose-dependent effect of *GRN* mutation on TDP-43 pathology and (b) timing as an important factor [37]. Therefore, it will be interesting to study TDP-43 aggregation with full or reduced PGRN expression. However, a recent examination of iPSC-derived cortical neurons from a CLN11 patient with *GRN* Thr272fsX10 homozygous mutation revealed increases of cytoplasmic TDP-43 and phospho-TDP-43 (p-TDP-43), as well as cleaved TDP-43 C-terminal fragments, compared with control cells [118]. Additionally, pathological 25-kDa C-terminal TDP-43 fragments have been detected in CLN11 patient-derived lymphoblastoid cell lines and their extracellular vesicles [119] (Table 1). These discrepancies require further clarification.

**Table 1:** TDP-43 pathology in human models of PGRN deficiency

Model	TDP-43 pathology	References
*Patient-derived models*		
CLN11 patient iPSC -derived cortical neurons with homozygous PGRN p.T272SfsX pathogenic variant	Increases of cytoplasmic TDP-43 and p-TDP-43, as well as cleaved TDP-43 C-terminal fragments	[118]
bvFTD patient iPSC-derived neurons with heterozygous PGRN p.S116X pathogenic variant	Higher redistribution of TDP-43 from the nucleus to the cytoplasm	[9]
FTD patient iPSC-derived cortical neurons with heterozygous *GRN* IVS1 + 5G > C splicing mutation	TDP-43 displays normal nuclear staining with no aggregates	[136]
FTD patient iPSC-derived cortical neurons with heterozygous PGRN p.R493X pathogenic variant	TDP-43 aggregates in the cytoplasm; mutant TDP-43 shows a doubled half-life	[56]
iPSC-derived cortical neurons from three FTD patients with heterozygous PGRN p.A9D pathogenic variant	Reduced nuclear TDP-43 and increased insoluble TDP-43	[45]
Lymphoblastoid cell lines (LCLs) from patients carrying a heterozygous PGRN p.T272SfsX10, p.C149LfsX10, p.Q341X, or IVS1-2A > G; or homozygous p.T272SfsX10 PGRN pathogenic variant	Increased p-TDP-43 levels in LCLs and EVs; 25-kDa fragments of TDP-43 are only present in patient LCLs	[119]
Monocyte-derived induced microglia (iMGs) from FTD patients with PGRN p.M1? and p.W147X pathogenic variant	Cytoplasmic aggregation of phosphorylated and ubiquitinated TDP-43; lipid droplet accumulation, lysosomal abnormalities, and impaired phagocytosis	[14]
FTD patient-derived fibroblasts with PGRN mutations p.M1? and p.W147X pathogenic variant	Cytoplasmic increases of p-TDP-43	[14]
*GRN silencing-based models*		
SH-SY5Y, HeLa and HEK293T cell lines with downregulation or upregulation of *GRN* by siRNA/overexpression, respectively	Cell line-specific accumulation of sarkosyl-insoluble TDP-43 in PGRN-deficient cells	[40]
Human neural progenitor cells from aborted female fetus; stable *GRN* silencing by shRNA	Increases of poly-ubiquitinated proteins	[113]
SH-SY5Y cells with stable *GRN* silencing by shRNA	TDP-43-positive intranuclear inclusions	[206]
Human primary skin fibroblasts from neurologically healthy donors, with transient *GRN* silencing by siRNA	Increased production of a 25 kDa TDP-43 C-terminal cleavage product	[205]
2D coculture model of neurons and astrocytes, with *GRN* knockout either in neurons or in astrocytes	Lack of overt TDP-43 pathology at 4th week of coculture; *STMN2* mis-splicing at 4th week of coculture, which can be rescued by recombinant PGRN	[11]
3D brain organoids composed of *GRN*^−/−^ or *GRN*^+/+^ neurons and astrocytes	TDP-43 mislocalization in both *GRN*^−/−^ astrocytes and *GRN*^+/+^ neurons, or *GRN*^+/+^ astrocytes and *GRN*^−/−^ neurons; cytoplasmic p-TDP-43 inclusions at 4th week of coculture; *STMN2* mis-splicing only in organoids containing PGRN-deficient astrocytes, independently of neuronal PGRN status	[11]
Cortical organoids transplanted with GRN^−/−^ induced astrocytes (iASTs)	Extranuclear accumulation of TDP-43 in neurons	[7]

The full TDP-43 pathology includes nuclear depletion, cytoplasmic hyperphosphorylated and ubiquitinated inclusions, and downstream loss-of-function phenotypes, such as cryptic splicing of *STMN2* and *UNC13a*

#### Molecular links between PGRN deficiency and TDP-43 pathology

The mechanisms for the presence of TDP-43 pathology in virtually all described cases of FTD-*GRN* remain unclear. PGRN deficiency leads to impaired autophagy, which is often associated with TDP-43 aggregation [36, 38]. The consequences of TDP-43 loss of function can vary depending on the cellular environment and the additional RNA-binding proteins, which can be expressed specifically in different tissues [195]. Notably, *Grn* overexpression can mitigate axon growth deficits induced by mutant TDP-43 or reduced expression of wild-type TDP-43 in a zebrafish model, indicating a connection between Pgrn and TDP-43 function [2]. Consistent with this observation, PGRN expression reduces the levels of insoluble TDP-43 in a mouse model harboring the TDP-43 disease-associated mutation p.A315T [196].

Despite these advancements, some questions regarding TDP-43 pathology in the context of *GRN* mutation still need to be investigated, such as the onset of TDP-43 pathology in *GRN* mutation carriers, particularly asymptomatic carriers, and the progression of TDP-43 pathology over time (Fig. 5). It is also essential to develop tools such as PET tracers for TDP-43 to aid in differential diagnosis, clinical trial design and evaluation of therapeutic strategies for TDP-43-related disorders [144].

## Modeling PGRN deficiency

In this section, we focus on the comparison of established disease hallmarks observed in patient brain tissues and biofluids with those observed in rodent and in vitro human models of PGRN deficiency.

### FTD-*GRN* patient brains versus Pgrn deficiency in mouse

While mouse models of Pgrn deficiency are crucial for understanding disease mechanisms and testing treatments, they do not fully recapitulate the entire spectrum of disease phenotypes and pathologies observed in FTD-*GRN* patients [8, 10].

Hemizygous (*Grn*^+/−^) mice “genetically corresponding” to FTD-*GRN* patients carrying one heterozygous mutation display only mild behavioral phenotypes and lack glial activation along with TDP-43 aggregates, as reviewed extensively [8, 10, 197, 198] (Fig. 1).

On the other hand, *Grn*^−/−^ mice “genetically correspond” to lysosomal storage disorders, such as CLN11 caused by homozygous *GRN* mutations. These animals recapitulate some of the key clinical and neuropathologic features of FTD-*GRN* in an age-dependent manner, including cognitive deficits (learning and memory), impaired social interactions, neuronal loss, microgliosis, astrogliosis, and TDP-43 proteinopathy [8, 10, 16, 35, 130, 131, 133, 134, 199–201]. However, *Grn*^−/−^ mice show relatively mild cortical atrophy compared with the usually pronounced, often asymmetrical atrophy observed in human FTD-*GRN* brains. Additionally, TDP-43 proteinopathy is more significant in the frontal cortex of patients than in animal models [7, 197]. In isolated cases, *Grn*-knockout mice do not develop a characteristic pattern of cortical neurodegeneration with TDP-43 pathology [202], but instead show an age-dependent retinal thinning phenotype with nuclear depletion of TDP-43, similar to that observed in FTD patients [202].

In addition, *Grn*^−/−^ mice present exaggerated inflammatory responses to a variety of stressors, such as toxins, infection or traumatic brain injury, as reviewed by [197].

With respect to behavioral phenotypes, selective Pgrn depletion in neurons or microglia in mice has different outcomes [116, 203, 204]. Neuronal *Grn* knockout leads to social dominance defects, whereas microglial Pgrn deficiency is associated with compulsive behaviors (i.e., excessive grooming) [116, 200, 204].

The incomplete recapitulation of human disease in mouse models might be due to the human-specific factors, such as differences in genetics, tissue-specific molecular mechanisms, or brain structure, connectivity, and function. Additionally, the shorter lifespan of rodents hinders the progression of FTD.

### Recapitulating TDP-43 pathology in human-derived models of PGRN deficiency

Human-derived in vitro 2D and 3D cellular models have been developed, either derived from FTD-*GRN* patients or by lowering *GRN* levels with small interfering RNA [40, 205] or short-hairpin RNA (shRNA) [113, 206] in human cells.

A currently debated question in the field is whether PGRN deficiency causes TDP-43 pathology through a cell-autonomous or non-cell-autonomous mechanism. Single-cell cultures modeling PGRN deficiency, reproduce only some aspects of TDP-43 pathology [9, 40, 45, 113, 118, 119, 136, 205, 206] (see Table 1), or these hallmarks have not been tested at all [57, 129, 207–210]. To date, only single-cell cultures of induced microglia reproduce all aspects of TDP-43 pathology [14].

As a logical follow-up to FTD-*GRN* patient-derived iPSCs, protocols for human brain organoids have been developed [11, 129, 210] (Table 1). These 3D tissue cultures possess ventricle-like structures that primarily recapitulate fetal cortical neurogenesis and the cell lineages involved [211, 212]. However, in terms of cortical architecture, organoids do not have the same layer-like (laminar) organization as the human brain does [213]. A lack of vascularization is thought to be associated with chronic cellular stress and limited growth due to limited exchange of oxygen/nutrients and brain detoxification [211, 212]. Importantly, human brain organoids derived from the iPSCs of patients carrying the p.Ser301Cysfs*61 PGRN variant in heterozygous or homozygous state present decreased dimensions compared with control organoids [210], further corroborating the trophic properties of PGRN.

In conclusion, the above-described human models of PGRN deficiency highlight the non-cell-autonomous mechanisms in the development of TDP-43 pathology and confirm *GRN*^−*/*−^ astrocytes as drivers of at least some aspects of TDP-43, although further studies are needed to obtain a more precise picture. For example, it is still not known whether FTD-*GRN* patient-derived human brain organoids recapitulate TDP-43 pathology.

## Effects of PGRN deficiency on neuronal and non-neuronal cells of the CNS

### Effects of PGRN deficiency on neurons in animal and human-derived models

Initially, the effects of PGRN deficiency were investigated mainly in neurons, cells that are progressively lost during neurodegeneration. Evidence highlights the potential role of PGRN in neuronal development, synaptic plasticity, maintenance, pruning, neurotransmission, and regulation of inflammation, as reviewed by [1, 8, 10].

PGRN has been detected in synaptic compartments, suggesting its involvement in synaptic functions [214]. Indeed, PGRN acts as a neuronal growth factor that promotes neurite sprouting and survival in cultured primary cortical and motor neurons and modulates both the number and the structure of synapses [214–217]. *Grn* knockout in mice and in rat primary hippocampal cultures led to alterations of neuronal morphology with decreased synaptic connectivity and plasticity [218, 219]. Importantly, synaptic dysfunction preceded microgliosis and lipofuscinosis in animal and cellular models of Pgrn deficiency [2, 218, 219]. The putative receptor through which PGRN exerts its neurotrophic properties remains to be identified [1]. Sortilin 1, a neuronal PGRN receptor whose splicing is regulated by TDP-43, has been shown to be involved [220, 221]. In human models, a recent study employing proteomics, lipidomics, and metabolomics confirmed that PGRN regulates neuroinflammation, neurite outgrowth, and purine metabolism [222]. Pathways altered by PGRN deficiency include neuron projection, synaptic dysfunction, and brain metabolism [222]. This study revealed that neurons are more susceptible to PGRN depletion than are iPSCs [222].

Regarding neuronal subpopulations affected by Pgrn deficiency, two different mouse models with Pgrn deficiency (Grn^-/-^ mice with deletion of exons 2–13 of the mouse *Grn* gene and *Grn* R493X knockin mice) demonstrated a significant loss of thalamic Foxp2^+^ excitatory neurons (> 20%) occurring from 12 to 19 months, compared to control animals [13, 131]. Interestingly, an earlier study reported that FOXP2^+^, CTIP2^+^, or TBR1-TUJ1^+^ neuronal clusters are underrepresented in iPSC-derived cortical neurons obtained from FTD-*GRN* patients carrying heterozygous *GRN* IVS1 + 5G > C mutation [136] (Table 1). In contrast, other iPSC-based neuronal models derived from FTD-*GRN* patients (p.S116X^+/−^, p.R418X^+/−^, and p.R493X^−/−^) yielded similar percentages of various neuronal clusters [9, 108](Table 1). It is worth noting that FOXP2 plays a crucial role in the development of speech and language, functions usually severely impaired in FTD-*GRN* patients. Moreover, *FOXP2* polymorphisms modulate verbal fluency in FTD patients [223].

The presented evidence raises the question of whether specific neuronal subpopulations are inherently more vulnerable to degeneration in PGRN deficiency.

#### Upregulation of WNT/beta-catenin signaling and its significance in neurodevelopmental signaling

In the context of PGRN deficiency and many other neurodegenerative diseases such as AD, PD and Huntington’s disease (HD) [224, 225], upregulation of the WNT/beta-catenin signaling pathway is commonly seen [224]. WNT/beta-catenin promotes the proliferation of neural progenitor cells (NPCs) and their differentiation into neurons in the developing cortex [226], regulates neuronal migration to their appropriate cortical layers and guides axonal growth [227]. WNT/beta-catenin is also crucial for adult stem cell proliferation and the growth of various tissue organoids, and its reactivation is crucial for healing after injury [228–230]. PGRN downregulates the WNT/beta-catenin signaling pathway in certain contexts, as reviewed by [230]. Accordingly, canonical and noncanonical WNT signaling activation is observed early in neuronal development, in human NPCs with decreased PGRN (approximately 50%) via shRNA [113], and in FTD patient-derived neuronal progeny on day 40 [136]. This phenomenon is also evident in adulthood, in FTD-*GRN* brains, in *Grn*^−/−^ mouse cortex, in peripheral cells from FTD-*GRN* patients, and in various cellular models [87, 113, 231, 232]. Importantly, a recent meta-analysis of transcriptomic data confirmed that the WNT signaling pathway is the most represented pathway in familial FTD caused by mutations not only in *GRN* but also in *C9orf72* [233].

Currently, there is an ongoing debate on the significance of these observations. While WNT upregulation could signify inherent dysfunction of neurodevelopmental signaling, its inhibition only partially rescues corticogenesis defects in patient-derived neurons [136]. WNT upregulation is more likely a compensatory response to PGRN deficiency (which persists throughout the lifetime), as WNT upregulation promotes neuronal survival in vitro, while its inhibition increases apoptosis in *Grn*-knockout mice [113].

### Microglial involvement: evidence from models and FTD-*GRN* patients

Microglia comprise < 10% of glial cells in the CNS. They are the resident immune cells [234] and the primary phagocytes in the brain, and function to clear pathogens, cellular debris, and misfolded proteins/aggregates [235–237]. Microglia sense neural and synaptic activity through neurotransmitter and neuromodulator receptors, and modulate neural activity through multiple mechanisms, including the release of cytokines and neurotrophic factors, and phagocytosis [238, 239]. PGRN, along with TREM2 and CX3CR1, serves as an immune checkpoint that suppresses aberrant microglial activation (reviewed in [240]). Indeed, PGRN deficiency promotes microglial transition to disease-associated microglia (DAMs) with specific gene and protein expression signatures [13–15, 22, 23, 44, 79]. Microglial activation has been detected by immunohistochemistry in Pgrn-deficient mouse brains and human induced microglia (iMGs) derived from FTD-*GRN* patients [13, 14, 16, 126, 130–132, 241], and by PET imaging using radiotracers that bind to TSPO (translocator protein) expressed by activated microglia [15, 19, 201, 242].

**Fig. 3:**
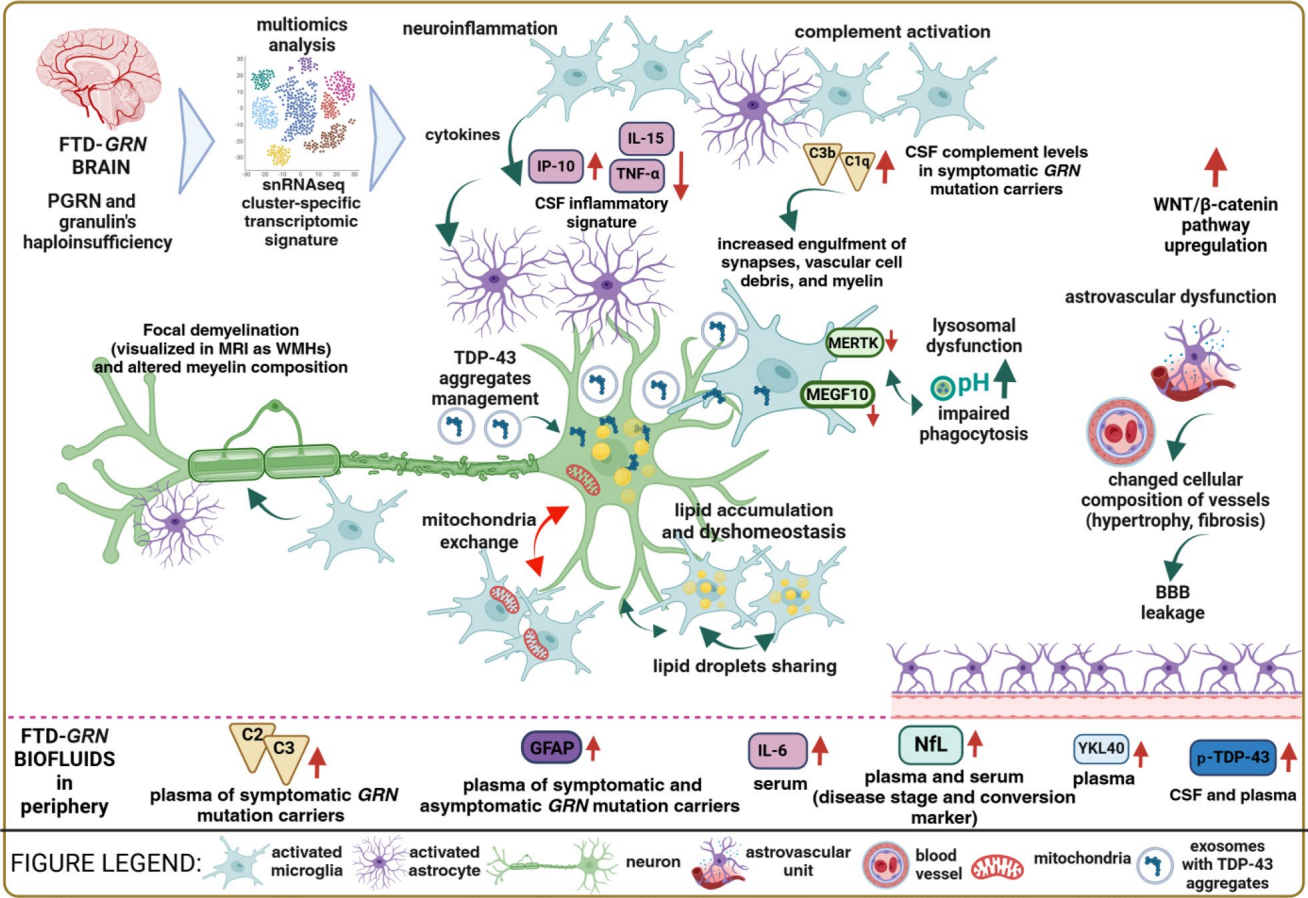
Complex interactions between neuronal and non-neuronal cells in the brain in FTD-*GRN*. Multiomics analyses of the transcriptome (bulk or snRNA-seq), metabolome, and proteome revealed that cell cluster-specific changes contribute to lysosomal dysfunction, TDP-43 pathology, neuroinflammation, gliosis, complement activation, synapse pruning, demyelination, and BBB alterations [7, 11, 12, 14, 56, 79, 115, 128] (upper panel). These changes are reflected by elevated CSF/plasma markers in FTD-*GRN* patients [23, 24, 91, 127, 243–245, 254, 255] (lower panel). The PGRN deficiency-associated lysosomal phenotype is characterized by a less acidic luminal pH [58] and altered proteolytic enzyme activity [40, 44, 57]. Microglia and astrocytes exhibit increased engulfment of synapses, vascular debris, myelin, and TDP-43 aggregates [7], accompanied by downregulation of phagocytic receptors (MEGF10 and MERTK) [11] and impaired phagocytic capacity [14]. The role of exosomes remains controversial, with evidence supporting both TDP-43 aggregate spread and disposal mechanisms [256, 257]. Focal demyelination in FTD-*GRN* brains correlates with disease severity and cognitive decline and has potential as an early marker of dysfunction in presymptomatic carriers of *GRN* mutations [18, 258]. Granulins are haploinsufficient in FTD-*GRN* patients and show therapeutic potential in PGRN deficiency mouse models [39, 44, 53]. BBB, blood‒brain-barrier; CSF, cerebrospinal fluid; C1q, complement C1q; C2, complement C2; C3b, complement C3b; PGRN, progranulin; FTD-*GRN*, frontotemporal dementia caused by *GRN* mutations; GFAP, glial fibrillary acidic protein; IP-10, interferon-γ-inducible protein-10; IL-6, interleukin 6; IL-15, interleukin 15; MEGF10, multiple EGF-like domains 10; MERTK, MER proto-oncogene; tyrosine kinase; MRI, magnetic resonance imaging; NfL, neurofilament light chain; pH, a scale of acidity; p-TDP-43, phosphorylated TDP-43; snRNA-seq, single-nucleus RNA sequencing; TDP-43, TAR-DNA binding protein 43; TNF-α, tumor necrosis factor α; WMHs, white matter hyperintensities; WNT/β-catenin; YKL40, chitinase 3 like 1

At the biofluid level, cytokine expression indicating CNS inflammation has been observed in the CSF and plasma of FTD-*GRN* patients [22]. Specifically, increased level of interferon-γ-inducible protein-10 and decreased levels of tumor necrosis factor α, IL-15, and RANTES (regulated on activation, normal T-cell expressed and secreted) have been detected in the CSF of FTLD patients carrying *GRN* mutations, compared with controls [243]. In addition, the serum interleukin 6 (IL-6) level is elevated in FTD-*GRN* compared with other genetic or sporadic forms of FTD [244] (Fig. 3). However, the IL-6 specificity for FTD-*GRN* has been challenged, as this marker cannot discriminate between sporadic and genetic FTD subtypes [245]. Recent research identified a panel of microglial activation markers (FABP3, MDH1, GDI1, CAPG, CD44, and GPNMB) in the CSF and microglia derived from FTD-*GRN* patients and a mouse model [23], which are potentially useful for monitoring microglial responses in clinical trials and therapy.

Microglia also play a role to support brain development and regulate synaptic plasticity in adulthood through synaptic pruning [246]. Synaptic pruning (peaking around the age of 2–4 years in the prefrontal cortex) eliminates redundant or less efficient synapses through phagocytosis to accommodate their initial overproduction [247, 248]. The involvement of complement proteins in microglia-mediated synaptic pruning was first reported in 2007 [249]. Complement proteins, notably complement C1q, mark synapses for removal during normal brain development [246, 249]. Aberrant or reactivated synaptic pruning precedes protein pathology in various neurodegenerative diseases [246, 250, 251]. To date, several mouse models of Pgrn deficiency have shown strong microglial activation with the involvement of the complement cascade [13, 15, 16, 44, 133]. In *Grn*^−/−^ mice, excessive C1qa-dependent pruning of inhibitory synapses in the ventral thalamus is observed, accompanied by hyperexcitability in the thalamocortical circuits and obsessive‒compulsive disorder-like grooming behaviors [16]. Notably, C1qa and C3 colocalize with lysosomes, suggesting inefficient degradation of these proteins [16]. In *Grn*^−/−^ mouse model, gene expression analysis showed that microglial activation is accompanied by endolysosomal dysfunction, TDP-43 pathology, and nuclear pore defects [13, 15]. However, the death rate of excitatory neurons is higher than that of inhibitory neurons in the *Grn*^−/−^ mouse thalamus at 19 months [13], which is in contrast with the previous observations [16]. Conditioned medium from Grn^−/−^ microglia has similar detrimental effects on neurons (such as promoting TDP-43 granule formation, nuclear pore defects, and cell death) as direct coculture of neurons with these microglial cells [13]. Postmortem immunocytochemistry confirmed the activation of microglia and astrocytes in FTD-*GRN* patient brains, along with abundant complement C1qa deposits particularly in the frontal cortex, increased inflammatory markers, and more pronounced gliosis in the frontal cortex than in the thalamus [7, 16].

Lui et al. proposed CSF and plasma complement proteins as FTD-*GRN* biomarkers since CSF C1qa and C3b levels increase gradually as the disease progresses [16]. A subsequent multicenter GENFI cohort study confirmed the utility of CSF and plasma complement proteins as biomarkers for presymptomatic and symptomatic FTD patients, including carriers of *GRN, C9orf72,* or *MAPT* mutations (*n* = 224) [24]. Indeed, CSF C1q and C3b, as well as plasma C2 and C3, are elevated in symptomatic mutation carriers compared with presymptomatic carriers and noncarriers [24] (Fig. 3).

#### Microglial lipid droplet formation in PGRN deficiency

Alterations of brain lysosomal lipid profiles have been observed in numerous models of PGRN deficiency. In the brains of *Grn*^−/−^ mice and FTD-*GRN* patients, accumulation of polyunsaturated triacylglycerides and saturated cholesteryl esters, as well as reductions of diacylglycerides and phosphatidylserines are observed [128] (Fig. 3). Lysosomal dysfunction, oxidative stress, microgliosis, lipofuscinosis and neuronal death accompanied by decreased bis(monoacylglycerol)phosphate levels and accumulation of glucosylsphingosine, are also observed in *Grn*^−/−^ mouse models [15, 58]. Treatment with recombinant PGRN rescued all these pathological markers in *Grn*^−/−^ mice and human iPSC-derived *GRN*^−/−^ microglia [58].

PGRN is a key regulator of lipid droplet formation in lipid droplet-accumulating microglia (LDAMs) [252]. As expected, *Grn*^−/−^ mouse microglia present with lipid droplet build-up, elevated ROS levels, and impaired phagocytosis, all indicative of accelerated aging [252, 253] (Fig. 3).

Finally, iMGs from FTD-*GRN* patients exhibit lipid droplet accumulation and impaired phagocytosis [14]. In addition, lysosomal abnormalities and neuroinflammation (complement C1q activation and upregulation of proinflammatory cytokines) were found to precede TDP-43 aggregation (Table 1). Further, lysosomal lipid biomarkers (glucosylsphingosine, GlcSph; ganglioside GM2 and globoside GB3) are increased in the plasma of *GRN* mutation carriers [91]. However, a recent study using single-cell lipid metabolic imaging platform highlighted increased newly synthesized lipid ratios with unchanged total lipids, suggesting a higher lipid turnover rate in *GRN*-knockdown human iPSCs and iMGs compared with control cells [129].

Taken together, microglial neuroinflammatory response plays a prominent role in FTD-*GRN*. Intriguingly, complement C1q treatment induces TDP-43 pathology in neurons and microglia, independent of the *GRN* status [13, 14]. C1qa and C3 deletion alleviates microglial toxicity, TDP-43 proteinopathy, and neuronal death [13, 16, 44]. However, loss of TREM2 rescues hyperactivation of microglia, but fails to attenuate lysosomal abnormalities or dyslipidemia in *Grn*-knockout mice, pointing to a primary role of lysosome deregulation in Pgrn deficiency [15]. Moreover, Grn/Trem2 double knockout mice display enhanced brain pathology, suggesting that the TREM2-dependent microglial hyperactivation plays a neuroprotective role in Pgrn deficiency [15].

### Oligodendrocyte dysfunction in FTD-*GRN*

Oligodendrocytes comprise approximately 45%–75% of glial cells. They produce and maintain myelin, allowing faster and more efficient transmission of nerve impulses [234]. Oligodendrocyte progenitor cells are precursors of myelinating oligodendrocytes and may also contribute to the clearance of cellular debris (including damaged myelin) and the remodeling of neural circuits in the developing and adult brains [259]. The loss or damage of myelin is referred to as focal demyelination. In MRI, myelin loss can be observed as areas of increased signal intensity, called white matter hyperintensities (WMHs).

In FTD-*GRN* patients, WMHs are often correlated with disease severity and cognitive decline [260–262] and are accompanied by increased neuroinflammation and microglial/astroglial activation in affected brain regions. To date, atypical WMHs in FTD-*GRN* patients have been associated with increased levels of neurofilament light chain (NfL), a biomarker of neuronal damage [258] (Fig. 3). Most importantly, WMHs have been proposed as an early marker of dysfunction in presymptomatic carriers of *GRN* mutations [174, 263]. However, evidence regarding white matter integrity in preclinical *GRN* carriers is inconsistent, likely due to the small study cohorts and inclusion of individuals at various stages relative to the clinical onset. Some studies did not report any abnormalities on tractography, while others indicated reduced connectivity, most often in the left uncinate fasciculus, the left inferior occipitofrontal fasculus and the genu of the corpus callosum [144]. Notably, the study with the largest sample size (52 asymptomatic *GRN* carriers) reported increased diffusivity in the internal capsule due to axonal or myelin damage [174].

Lipidomic analyses have also detected alterations in myelin-building lipids in FTD-*GRN* brains and plasma [18, 258], including decreased sphingolipid (sulfatide, galactosylceramide, sphingomyelin) and myelin proteins in the frontal white matter, increased acylcarnitine levels in the frontal gray matter, and substantial accumulation of cholesterol esters in both frontal and parietal white matter, suggesting the breakdown of myelin [18]. An overactive breakdown of myelin lipids can act as a catalyst for gliosis and neurodegeneration in FTD-*GRN* (Fig. 3).

In summary, more evidence is needed to clarify how oligodendrocytes contribute to the FTD-*GRN* pathology. Proteomic analysis suggested that demyelination and neuronal loss occur at late stages in the brains of *Grn*^−/−^ mice (19 months of age) [17]. In addition, despite the pronounced myelin loss, cell density of mature oligodendrocytes is not reduced in frontal white matter of FTD-*GRN* patients [18]. However, increases of specific lysosphingolipids (glucosylsphingosin and lysosphingomyelins) in plasma of presymptomatic and symptomatic *GRN* mutation carriers suggest that early lysosomal dysfunction leads to deregulation of myelin maintenance and turnover [258].

### Complex response of astrocytes, microglia, and neurovascular cells to PGRN deficiency: another level of complexity added by snRNAseq analyses

Recent studies have implicated astrocytes and vascular cells in PGRN deficiency-related neurodegeneration [11, 12]. This finding was somewhat surprising, as resting astrocytes normally express low levels of PGRN.

Astrocytes constitute 19%–40% of the glial cell population [234]. Their specialized endfeet encompass the entire vascular system within the CNS [264], contributing to the formation of BBB alongside endothelial cells, vascular smooth muscle, pericytes, and the vascular basement membrane. They also play a vital role in the glymphatic system and are functionally and structurally interconnected with the BBB [264, 265]. The BBB regulates the passage of substances between the bloodstream and brain tissue, whereas the glymphatic system is primarily responsible for the circulation of CSF through the perivascular spaces in the CNS and the nocturnal elimination of brain waste products, including protein aggregates [265, 266]. In addition to providing metabolic and trophic support [264, 267–269], astrocytes control various aspects of neuronal functions, such as synaptic formation, transmission and plasticity, maintenance of ion homeostasis and release of gliotransmitters [270–272]. They recycle neurotransmitters such as glutamate and gamma-aminobutyric acid (GABA) from the synaptic cleft, preventing neurotransmission imbalances (i.e., glutamate overload and excitotoxicity). Astrocytes can also regulate cholesterol and sphingolipid metabolism [273]. Finally, astrocytes mediate synaptic pruning during development and in certain contexts in adulthood through multiple EGF-like domains 10 (MEGF10) and MER protooncogene, tyrosine kinase (MERTK) pathways [274, 275].

Initial studies on the role of astrocytes in PGRN deficiency revealed particularly severe astrocytosis, more extensive than microgliosis, and stronger axonal injury, in TBI mice with PGRN deficiency than *Grn*^+/+^ TBI animals [29, 276]. Administration of recombinant PGRN pre- or post-injury ameliorated pro-inflammatory astrocytic activation and reduced brain damage [29].

In a recent GENFI study correlating gene expression with gray matter atrophy patterns, regions of atrophy presented increased expression of astrocytic and endothelial gene clusters, whereas less affected regions presented increased expression of genes linked to neurons and microglia. These results suggest that astrocytes and endothelial cells play a role in the onset of neurodegeneration in FTD-*GRN* patients [115].

In addition, the plasma level of glial fibrillary acidic protein (GFAP), an established astrocytic marker, is significantly increased in asymptomatic [91] and symptomatic *GRN* mutation carriers [91, 277] (Fig. 3). Neuroimaging and biomarker analysis in presymptomatic *GRN* mutation carriers revealed positive correlations of several complement proteins with GFAP and NfL levels and negative correlations with gray matter volume in FTD-relevant brain regions [24]. NfL, a cytoskeletal scaffold protein released by neurons into the CSF and blood upon damage, can serve as a biomarker for identifying individuals at risk of progressing from the presymptomatic stage to the symptomatic stage of genetic FTD [278]. Another primary astrocytic activation marker, YKL-40 (also known as CHI3L1), has also been found to be elevated in the CSF or plasma of *GRN* mutation carriers [91, 127] (Fig. 3).

In a recent snRNAseq study, early deregulation of neurovascular cells, including astrocytes, endothelial cells, and pericytes, was observed in FTD-*GRN* brains [12]. Two major astrocyte subtypes were revealed with opposing levels of GFAP. The subtype with low GFAP expression (and high SLC1A2 and CABLES1) is associated with gray matter, whereas the GFAP-enriched cluster (and SLC38A1) is identified in cortical layer 1 and the white matter. FTD-*GRN* patients and controls show significant differences in the distribution of gray matter astrocyte subclusters and cellular composition of vessels [12]. Compared to the controls, FTD-*GRN* patients present increased numbers of fibroblasts and mesenchymal cells, reduced capillary coverage by pericytes, hypertrophic vascularization, and increased perivascular T cells in the brain. Additionally, the ratio of extracellular matrix protein (fibronectin) to endothelial tight junction protein (CLDN5) is higher in patients than in healthy controls, suggesting increased fibrosis and BBB alterations (Fig. 3). In addition, levels of GFAP, AQP4, and WDR49 are increased in the frontal cortex of FTD-*GRN* patients compared with controls, which correlate with neuronal loss and TDP-43 pathology. However, this study did not demonstrate overt microglial activation or complement upregulation [12].

In contrast, another recent comparative analysis of the thalamus and frontal cortex of 19-month-old Pgrn-deficient mice and FTD-*GRN* patients revealed microgliosis, astrogliosis, and complement activation in response to PGRN deficiency. In particular, astroglial pathology is highly conserved, characterized by upregulation of *GJA1, AQP4,* and *APOE* and downregulation of the glutamate transporter *SLC1A2* [7]. Immunofluorescence staining revealed activation of microglia and astrocytes in FTD-*GRN* brains in proximity to TDP-43 aggregates. The activated microglia and astrocytes contained significantly more synapses (PSD95^+^) in their processes than did those in controls, along with vascular cell debris (CD34^+^), myelin (MBP^+^), and TDP-43 fragments, suggesting increased phagocytic activity (Fig. 3). The engulfed synapses colocalized with the LAMP1 marker, indicating that they are removed by lysosomes [7]. Another study demonstrated that neurons cultured with hiPSC-derived astrocytes carrying a homozygous *GRN* R493X^−/−^ knock-in mutation showed significantly delayed spiking activity compared with neurons cultured with WT astrocytes. This effect was associated with increased GABAergic (GAD67^+^) and decreased glutamatergic synaptic markers (vGLUT1^+^) on day 30 [279]. On day 70, however, only the imbalance in the number of glutamatergic synapses persisted in cocultures of WT neurons with *GRN* R493X^−/−^ astrocytes [279].

Another difference revealed by bulk RNA sequencing of 3D heterotypic *GRN*^−/−^ organoids compared with control organoids is the decreased expression of phagocytosis receptors MERTK, MEGF10, and AXL (AXL receptor tyrosine kinase) [11] (Table 1 and Fig. 3). At the functional level, *GRN*^−/−^ astrocytes present significant defects in synaptosome phagocytosis that could not be rescued by recombinant PGRN [11]. This may also reflect glial activation. Indeed, neurotoxic reactive astrocytes induced by activated microglia lose the ability to engulf synapses and have decreased expression of the phagocytosis receptors MEGF10 and MERTK [280].

Overall, these findings indicate that PGRN deficiency strongly alters the functions of astrocytes, microglia, and the brain vasculature, which in turn affect neuronal performance.

However, there are also some controversies, such as the increased versus decreased phagocytic capacity [7, 11] or the types of neurons/synapses degenerating in Pgrn-deficiency models (excitatory vs inhibitory neurons) [13, 16]. These discrepancies may be due to the use of different models.

#### Potential mechanisms of CNS barrier deregulation in PGRN deficiency

**Fig. 4:**
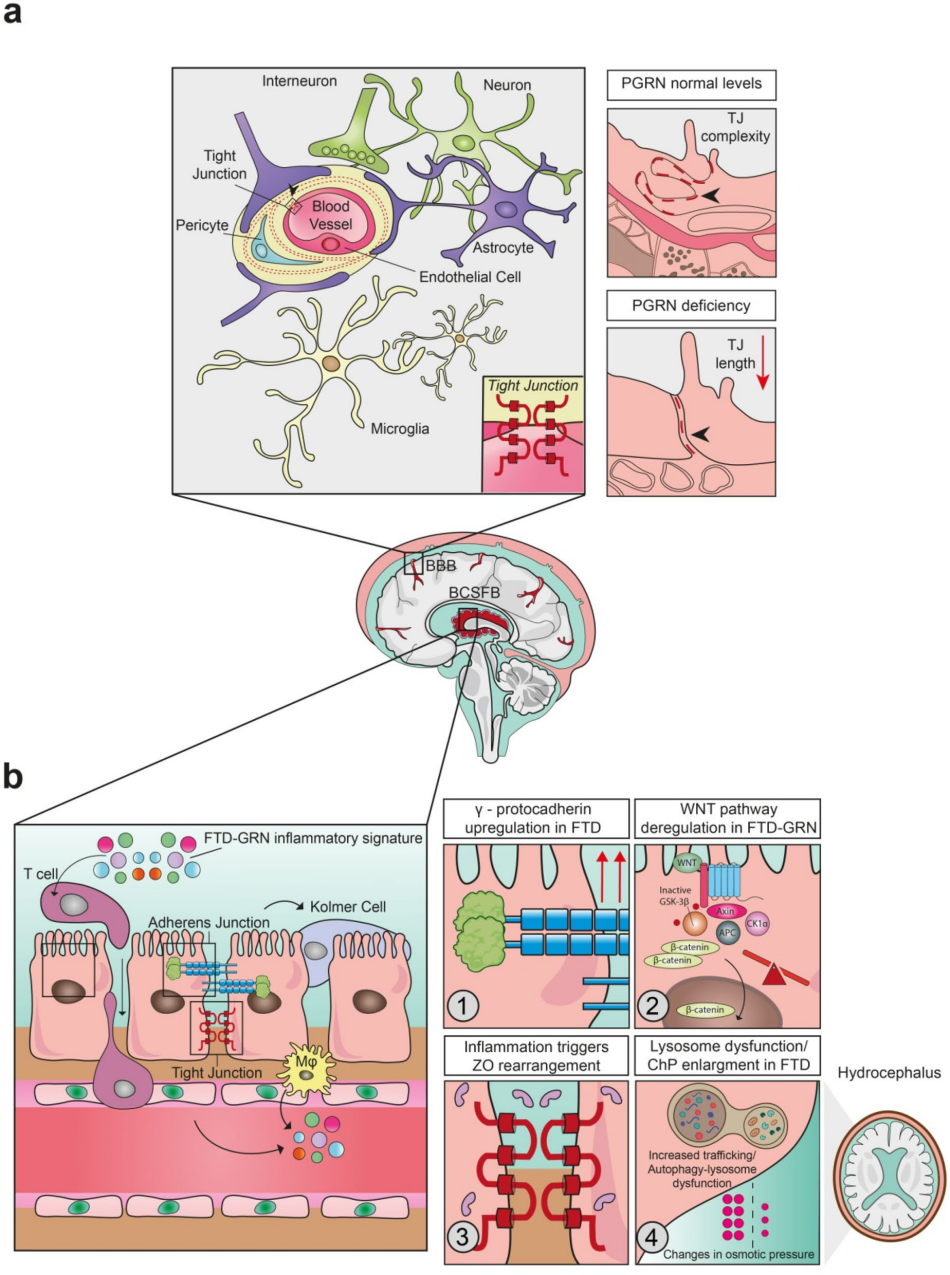
CNS barrier neuropathology induced by PGRN deficiency in FTD: documented (**a**) and speculative (**b**) mechanisms.** a** Left: illustration of BBB structure. Right: in mouse models of Pgrn deficiency, the BBB structural composition, particularly the tight junctions (TJs) between endothelial cells, become shorter and less convoluted, suggesting a weakened connection between neighboring endothelial cells [28]. **b** Left: activation of ChP epithelial cells by peripheral cytokine storms and immune cells, including T cells, Kolmer cells, and macrophages (M$\upvarphi$), promotes the release of chemokines into the stroma and cerebrospinal fluid (CSF), inducing changes in CSF composition and enabling the migration of peripheral immune cells to and from the CSF [295]. Right panel: four putative mechanisms leading to ChP dysfunction in FTD-*GRN*. ①The increased expression of γ-protocadherin [291], which forms adherens junctions, is a unique mechanism observed in FTD [289]. ②Upregulation of the WNT signaling pathway observed in FTD-*GRN* [136, 224, 231], may disrupt ChP [292]. ③The inflammatory milieu specific to FTD-*GRN* [24, 127] can potentially impair the ability of ZO proteins to assemble and form tight junctions. ④Dysfunction of the BCSFB observed in rare lipid storage diseases [293] suggests that PGRN may affect ChP secretory activity through its role in the regulation of lysosomal activity and lipid storage, thereby causing hydrocephalus. BCSFB, blood-CSF barrier; ChP, choroid plexus; CSF, cerebrospinal fluid; PGRN, progranulin; TJs, tight junctions; ZO, zonula occludens

The BBB and the BCSFB or plexus-vascular barrier regulate the exchange of substances between the CNS and the periphery [281]. Both the BBB and the BCSF are comprised of specialized endothelial cells, yet they display distinct permeability characteristics. In the BBB, tight junctions (TJs) are formed between endothelial cells, whereas the BCSF features a fenestrated endothelium, with TJs located between epithelial cells. While this represents the historically accepted definition, recent findings suggest that the permeability of the BCSF endothelium can be modulated in response to the integration and processing of peripheral environmental stimuli [282]. The BBB is highly impermeable, while the BCSFB, located within the ChP, allows for active transport and secretion of substances like water, electrolytes, glucose, and proteins, regulating CSF production, solute clearance, and removal of harmful substances, including toxins and drugs (Fig. 4) [283]. The ChP, composed of capillaries covered by epithelial cells, also interacts with glial cells in the glymphatic system to exchange CSF and interstitial fluid, aiding in solute clearance and maintaining a healthy brain environment [284].

Dysfunction of these barriers has been implicated in several neurodegenerative diseases [285–287], including FTD-*GRN* [12, 115]. Deregulation of the BBB in PGRN deficiency may be caused by dysfunction of vascular endothelial cells and astrocytes [12, 115]. Moreover, studies in mouse models of ischemia and TBI have shown that PGRN deficiency exacerbates BBB disruption, resulting in larger infarcts compared to control animals [28, 29]. Shorter, fewer, and less interlocking TJs in *Grn*^−/−^ mice suggest a weaker seal between endothelial cells [28](Fig. 4a).

FTD-*GRN* patients exhibit specific CSF signatures, including increased levels of p-TDP-43 [254, 255]. Changes in the cytokine milieu can be detected by the epithelial barrier, leading to alterations in TJs and promoting the migration of circulating immune cells [288] (Fig. 4).

Although there is no direct evidence linking BCSFB dysfunction to clinical symptoms in FTD-*GRN*, recent findings suggest it may play a relevant role. For example, characterization of ChP alterations in AD, such as epithelial atrophy, stromal fibrosis, vascular thickening, and TJ alterations, has provided insights into how barrier dysfunction may manifest in neurodegeneration [286, 289, 290]. While transcriptomic studies have shown overlapping genetic profiles between ChP tissues from FTD, AD, and HD patients, the upregulation of γ-protocadherin, a protein involved in regulating CSF secretion and immune dynamics at the apical side of the ChP epithelium, has been uniquely reported in FTD [289, 291] (Fig. 4b, zoom 1). Overactivation of WNT signaling reported in FTD-*GRN* [136, 224, 231] may also lead to ChP disruption [292] (Fig. 4b, zoom 2). In addition, increased ChP volume observed across different FTD subtypes (bvFTD, PPA, CBS, and PSP) has been associated with serum NfL levels, cognitive decline, and cortical atrophy, supporting the ChP dysfunction in disease progression [287] (Fig. 4b, zoom 4). These findings suggest that the ChP volume could be a potential biomarker for FTD-*GRN*. Moreover, in patients with lipid storage disorders such as Niemann-Pick disease, ChP epithelial cells show increased inflammatory gene expression, autophagosome accumulation and changes in hydrostatic pressure that potentially influence CSF dynamics [293] (Fig. 4b, zoom 4). Additionally, increased prevalence of idiopathic normal pressure hydrocephalus observed among bvFTD patients, suggests that altered CSF flow and pressure may play a role in the disease [294]**.** While the above-proposed mechanisms could be involved in PGRN deficiency, further studies are needed to clarify whether barrier alterations represent a pathological feature or are secondary to neuroinflammation in FTD-*GRN* patients.

## Bidirectional communication between neurons and glia

Neurons, as terminally differentiated cells, need to outsource many processes to adjacent glia to guarantee brain survival throughout their lifetime [296]. While metabolic interplay or neurotransmission between neurons and glia have been relatively well described [297], the trafficking modes of organelles, protein aggregates or lipids remain poorly understood. In this section, we therefore consider how neurons and glia may cooperate to maintain brain homeostasis through the exchange of organelles containing products of cellular metabolism and how the deregulation of these processes may aggravate PGRN-related neurodegeneration.

### Bidirectional transfer of mitochondria

Mitochondria are essential for meeting the high energy demands of the brain. Neurons, which cease dividing shortly after birth, must maintain effective mechanisms to repair and replenish damaged mitochondria exposed to oxidative stress [298]. This involves the selective degradation of damaged mitochondria through mitophagy, alongside mitochondrial biogenesis and dynamics, collectively forming a quality control system crucial for cellular energy homeostasis [299, 300]. Recent research has highlighted that mitochondria can be transferred bidirectionally between donor and recipient cells for degradation or to increase survival (reviewed by [301]). Davis et al. coined the term “transmitophagy”, describing the transfer of damaged mitochondria from retinal neurons to adjacent astrocytes for disposal [302]. This phenomenon has subsequently been reported by other groups [296] in AD [303] and PD [304]. The opposite phenomenon also occurs: astrocytes or microglia can send functional mitochondria to damaged neurons to promote their survival [305, 306] (Fig. 3).

To date, studies in cellular and animal models have demonstrated that PGRN deficiency influences mitochondrial biology [307–309]. PGRN facilitates mitophagy [310], and PGRN deficiency leads to downregulation of parkin, a key mitophagy regulator, as well as parkin downstream targets, mitofusin 2 (MFN2) and voltage-dependent anion channel 1 (VDAC1), in control fibroblasts with *GRN* silencing [205]. The observed effects of PGRN deficiency on mitochondrial biology and mitophagy may be indirect and mediated by TDP-43 pathology, which is well known to disrupt mitochondrial function [178, 205]. Recently, a direct mechanism by which PGRN modulates mitochondrial dynamics and complement activation has been described [309]. In the retinal pigment epithelium of *Grn*^−/−^ mice, loss of mitochondrial fission protein 1 leads to mitochondrial hyperfusion and bioenergetic defects, followed by NF-kB-dependent activation of complement C3a receptor signaling, resulting in retinal inflammation [309]. In this model, mitochondrial dysfunction and microglial activation could be rescued by C3aR antagonists.

However, whether there are other mechanisms through which PGRN deficiency directly or indirectly affects mitochondrial function in different cell types in the brain remains an open question. It can be expected that glia with compromised mitochondria or their removal system cannot support neurons through bidirectional transfer of these organelles.

### Removing TDP-43 aggregates from the brain: interaction between glial and non-glial cells

Recent studies have also highlighted the importance of coordinated interactions between astrocytes and microglia in protein aggregate clearance, with each cell type performing distinct functions [27, 311]. Protein aggregates can be transferred between neurons and glia as free molecules, within extracellular vesicles (EVs), or through tunneling nanotubes (TNTs) as free aggregates or within lysosomal-derived vesicles [27]. Transfer from neurons to glia with subsequent glial phagocytosis has been documented for protein aggregates/intermediates (α-synuclein, beta-amyloid, and tau) [27, 240, 306, 311]. α-Synuclein promotes TNT formation between neurons and microglia, preferentially at sites of toxic accumulation [306]. Sharing of the toxic α-synuclein burden by microglia attenuates the inflammatory microglial profile and relieves neurons [306, 312]. The removal of intracellular TDP-43 aggregates involves the phagocytosis‒autophagy axis and the ubiquitin‒proteasome system [25, 26]. However, currently, the specific mechanisms of TDP-43 aggregate removal in different cell types and whether there is a hierarchy of mechanisms/modalities remain unknown [7, 14, 306, 313]. Another important point to consider is the presence of comorbid proteinopathies (tau and FUS) that increase with age in FTD-*GRN* patients [180, 314, 315]. In a mouse model of tauopathy, Pgrn reduction increases tau and α-synuclein inclusions, leading to decreased survival rate and worsening of disinhibited behavior [316]. In an AD mouse model, selective depletion of microglial Pgrn impairs β-amyloid phagocytosis, increases plaque burden, and exacerbates cognitive deficits [317]. These results highlight the important role of PGRN in protein aggregate clearance.

Among the different modalities of intercellular communication, EVs, particularly exosomes, play a significant role in health and disease [318–320]. Exosomes are secreted by various cell types, such as neurons, astrocytes, microglia, and oligodendrocytes, and carry bioactive molecules such as proteins, lipids, and nucleic acids (including DNA, mRNAs, and microRNAs) [319, 321]. To date, both TDP-43 and p-TDP-43 have been shown to be carried by exosomes and microvesicles [322] (Fig. 3). Exosomes seem to play a protective role against TDP-43 accumulation [256]. On the other hand, they may also contribute to the spread of pathology [257, 323]. Further studies are needed to determine their precise role in reducing the TDP-43 burden in neurons versus transferring pathology to other cell types.

With respect to EV production, initial report demonstrated that fibroblasts from FTD-*GRN* patients secrete a reduced quantity of EVs compared with controls. However, *GRN* knockdown in SH-SY5Y cells promoted the secretion of EVs [324]. Arrant et al. hypothesized that lysosomal dysfunction associated with PGRN deficiency could lead to compensatory increases in the secretion of exosomes from the endolysosomal compartment [325]. Indeed, symptomatic (but not asymptomatic) FTD-*GRN* patients present elevated levels of brain and plasma EVs, a phenomenon that was also corroborated in the brains of *Grn*^−/−^ mice following the onset of pathology [325]. Specifically, *Grn*^−/−^ mice present altered EV protein content with increased levels of two proteins enriched in astrocytes, i.e., excitatory amino acid transporter 2, which removes the neurotransmitter glutamate from the synaptic cleft, preventing its excessive accumulation, and Na^+^/K^+^-ATPase, compared with those in control animals, reflecting the occurrence of astrocytosis at that stage [325]. Exosomes can also play both pro-neuroinflammatory and pro-regenerative roles in CNS dysfunction [319, 326], but their role in FTD-*GRN* has yet to be determined. Finally, exosomes derived from the CSF or blood of FTD-*GRN* patients, especially their miRNA cargoes, are potential biomarkers for early diagnosis and monitoring of progression. In the GENFI study, the levels of miR-204-5p and miR-632 are significantly lower in CSF exosomes derived from symptomatic carriers of genetic mutations than in those derived from presymptomatic carriers [327].

## Conclusions

Recent snRNAseq studies have highlighted the critical role of neuronal and glial diversity in the formation of specific neuronal networks and brain function [328–330]. PGRN deficiency affects virtually all CNS cell clusters (Fig. 3). The main hallmarks of FTD-*GRN*, such as lysosomal deregulation, protein and lipid dyshomeostasis, neuroinflammation, demyelination, and synapse dysfunction, manifest in all these cell populations albeit with different degrees. The phenotypes of DAM and LDAM in PGRN-deficient microglia are being characterized with greater precision.

Studies in FTD-*GRN* patient brains, along with modeling PGRN deficiency in 2D co-cultures and 3D engineered brain organoids, highlight the emerging role of astrocytes in TDP-43 pathology. In addition, increased levels of GFAP in presymptomatic *GRN* mutation carriers suggest early deregulation of astroglia in the disease course. Concomitant alterations of astrocytes, endothelial cells, and pericytes lead to compromised integrity of brain barriers, such as BBB. The role of oligodendrocytes in the progression of FTD-*GRN* is being preliminarily delineated.

**Fig. 5:**
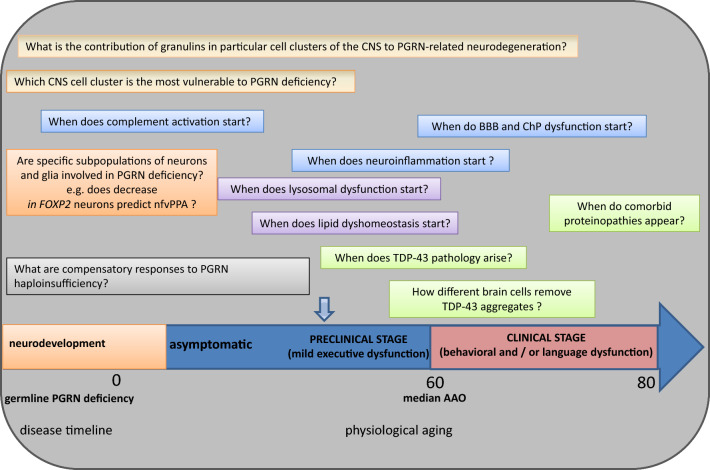
Outstanding questions that highlight unresolved issues, i.e., the sequence of pathological events in FTD-*GRN* or CLN11 patients and compensatory responses to PGRN deficiency. The answers to these questions may differ depending on PGRN dosage and life stage. BBB, blood–brain barrier; ChP, choroid plexus; CNS, central nervous system; PGRN, progranulin; nfvPPA, non-fluent variant of primary progressive aphasia

Questions regarding PGRN deficiency in FTD-*GRN* patients and disease models remain to be answered (Fig. 5).

We are only starting to grasp how PGRN deficiency impacts the bidirectional exchange of organelles and metabolic products, like TDP-43 protein aggregates and lipids, between glia and neurons.

Germline *GRN* mutations initiate complex cascades that lead to clinical symptoms after several decades. The final landscape of the cellular/molecular changes observed in the CNS/biofluids of FTD-*GRN* patients substantially overlaps with that of many other neurodegenerative diseases (Fig. 3). In addition to CSF/plasma markers (NfL, GFAP, YKL40, p-TDP-43, complement proteins, and IL-6) (Fig. 3) that reflect neurodegenerative and neuroinflammatory changes occurring in the CNS of FTD-*GRN* patients, the biomarker potential of other astrocytic and neuronal proteins, such as WDR49, RPH3A, NPTX2, and NEFM, requires further validation. Although most of the proposed biofluid markers of FTD-*GRN* overlap with those of other neurodegenerative diseases, they may be used in combination with specific clinical phenotypes and decreased PGRN levels. Additionally, the roles of systemic inflammation and the gut microbiome need to be determined.

Although PGRN is postulated to play a role in embryogenesis [331], the effects of its deficiency on neurodevelopmental processes, such as corticogenesis, await investigations in adequate models. It seems that integrating data from FTD-*GRN* and CLN11 patients and various models of PGRN deficiency (animal and cellular) will be essential to gain a comprehensive understanding of disease development, progression, and possible new therapeutic avenues.

## Supplementary Information

**Additional file 1**. **Table S1**. Comparison of clinical features and early manifestations in patients with FTD-*GRN* versus typical manifestations of behavioral variant frontotemporal dementia (bvFTD), non-fluent (nfvPPA) and logopenic variants of primary progressive aphasia (lvPPA), as well as the atypical clinical phenotype described in carriers of homozygotic *GRN* mutations

## Data Availability

Not applicable.

## Abbreviations

AD: Alzheimer’s disease
ALS-FTSD: Amyotrophic lateral sclerosis-frontotemporal dementia spectrum disorder
ATP: Adenosine triphosphate
BBB: Blood‒brain barrier
BCSFB: Blood–cerebrospinal fluid barrier
bvFTD: Behavioral variant of frontotemporal dementia
C9orf72: Chromosome 9 open reading frame 72
ChP: Choroid plexus
CLN11: Neuronal ceroid lipofuscinosis 11
CNS: Central nervous system
CSF: Cerebrospinal fluid
CTIP2: COUP-TF-interacting protein 2
FTD: Frontotemporal dementia
FTLD: Frontotemporal lobar degeneration
GABA: Gamma-aminobutyric acid
GFAP: Glial fibrillary acidic protein
HD: Huntington’s disease
IL-6: Interleukin 6
iMG: Induced microglia
iPSCs: Induced pluripotent stem cells
MEGF10: Multiple EGF-like domains 10
MERTK: MER proto-oncogene, tyrosine kinase
MRI: Magnetic resonance imaging
NfL: Neurofilament light chain
nfvPPA: Non-fluent variant of primary progressive aphasia
NPCs: Neural progenitor cells
PD: Parkinson’s disease
PET: Positron emission tomography
PPA: Primary progressive aphasia,
PSAP: Prosaposin
PSP: Progressive supranuclear palsy
shRNA: Short hairpin RNA
SLPI: Secretory leukocyte protease inhibitor
TDP-43: TAR-DNA binding protein 43
TJ: Tight junction
TMEM106B: Transmembrane protein 106B
WMHs: White matter hyperintensities
