# Supplementary material for: Progranulin deficiency in the brain: the interplay between neuronal and non-neuronal cells
Source: Transl Neurodegener. 2025 Apr 16;14:18. doi: 10.1186/s40035-025-00475-8 (PMC12001433; doi:10.1186/s40035-025-00475-8)
Supplement: Supplementary file 1 — Additional file 1. Table S1. Comparison of clinical features and early manifestations in patients with FTD-GRN versus typical manifestations of behavioral variant frontotemporal dementia (bvFTD), non-fluent (nfvPPA) and logopenic variants of primary progressive aphasia (lvPPA), as well as the atypical clinical phenotype described in carriers of homozygotic GRN mutations [file 40035_2025_475_MOESM1_ESM.docx]

Supplementary data

Table S1. Comparison of clinical features and early manifestations in patients with FTD-*GRN* versus typical manifestations of behavioral variant frontotemporal dementia (bvFTD) ([1](#_ENREF_1)), non-fluent (nfvPPA) and logopenic variants of primary progressive aphasia (lvPPA) ([2](#_ENREF_2)) as well as the atypical clinical phenotype described in carriers of homozygotic *GRN* mutations ([3](#_ENREF_3)).

| **bvFTD core features** | **Clinical characteristics of FTD-*GRN* with bvFTD phenotype in comparison to *MAPT* and *C9orf72* phenotype** | **Early manifestation in heterozygous**  ***GRN* mutation carriers prior to the onset of clinical symptoms** | **Possible pronounced features in carriers of homozygotic *GRN* mutations** |
| --- | --- | --- | --- |
| Behavioural disinhibition | very pronounced especially in FTD-*MAPT* |  | yes |
| Apathy or inertia | very pronounced in FTD-*GRN* |  | yes |
| Loss of sympathy and empathy | more frequent in FTD-*MAPT* |  | yes |
| Perseverative, stereotyped or compulsive/ritualistic behaviour | more frequent in FTD-*MAPT* |  | yes |
| Hyperorality and dietary changes | more frequent in FTD-*MAPT* |  | yes |
| Neuropsychological profile: executive/generation deficits with relative sparing of memory and visuospatial functions | in FTD-*GRN* visuospatial function and memory may be also affected | early attention and executive deficits, slowed mental processing speed, impaired facial emotion recognition | executive dysfunction accompanied by possible severe visual impairment |
| **nfvPPA core features** | **clinical characteristics of FTD-*GRN* with PPA phenotype** |  |  |
| Agrammatism | present in PPA-*GRN* |  |  |
| Apraxia of speech | uncommon in PPA-*GRN* |  |  |
| **lvPPA core features** | **clinical characteristics of FTD-*GRN* with PPA phenotype** |  |  |
| Anomia | present in PPA-*GRN* | impaired phonemic fluency and phonological processing |  |
| Impaired repetition of sentences / phrases | present in PPA-*GRN* |  |  |
| **Other features** |  |  |  |
| Psychotic symptoms | rare in FTD-*GRN*; much more common in *C9orf72* (mainly somatoform delusions) |  | visual hallucinations |
| Motor symptoms | possible parkinsonism later in the disease course |  | cerebellar ataxia  epilepsy |

References:

1. Rascovsky K, Hodges JR, Knopman D, Mendez MF, Kramer JH, Neuhaus J, et al. Sensitivity of revised diagnostic criteria for the behavioural variant of frontotemporal dementia. Brain. 2011 Sep;134(Pt 9):2456-77.

2. Gorno-Tempini ML, Hillis AE, Weintraub S, Kertesz A, Mendez M, Cappa SF, et al. Classification of primary progressive aphasia and its variants. Neurology. 2011 Mar 15;76(11):1006-14.

3. Huin V, Barbier M, Bottani A, Lobrinus JA, Clot F, Lamari F, et al. Homozygous GRN mutations: new phenotypes and new insights into pathological and molecular mechanisms. Brain. 2020 Jan 1;143(1):303-19.
